# Alterations in the Proteome and Phosphoproteome Profiles of Rat Hippocampus after Six Months of Morphine Withdrawal: Comparison with the Forebrain Cortex

**DOI:** 10.3390/biomedicines10010080

**Published:** 2021-12-31

**Authors:** Hana Ujcikova, Adam Eckhardt, Lucie Hejnova, Jiri Novotny, Petr Svoboda

**Affiliations:** 1 Laboratory of Membrane Receptors, Department of Neurochemistry, Institute of Physiology of the Czech Academy of Sciences, Videnska 1083, 142 20 Prague 4, Czech Republic; svobodap@fgu.cas.cz; 2 Laboratory of Translational Metabolism, Institute of Physiology of the Czech Academy of Sciences, Videnska 1083, 142 20 Prague 4, Czech Republic; adam.eckhardt@fgu.cas.cz; 3 Department of Physiology, Faculty of Science, Charles University, Vinicna 7, 128 43 Prague 2, Czech Republic; lucie.hejnova@natur.cuni.cz (L.H.); jiri.novotny@natur.cuni.cz (J.N.)

**Keywords:** protracted morphine withdrawal, rat hippocampus, rat brain cortex, gel-based proteomics, energy metabolism, oxidative stress, nLC-MS/MS

## Abstract

The knowledge about proteome changes proceeding during protracted opioid withdrawal is lacking. Therefore, the aim of this work was to analyze the spectrum of altered proteins in the rat hippocampus in comparison with the forebrain cortex after 6-month morphine withdrawal. We utilized 2D electrophoretic workflow (Pro-Q^®^ Diamond staining and Colloidal Coomassie Blue staining) which was preceded by label-free quantification (MaxLFQ). The phosphoproteomic analysis revealed six significantly altered hippocampal (*Calm1*, *Ywhaz*, *Tuba1b*, *Stip1*, *Pgk1*, and *Aldoa*) and three cortical proteins (*Tubb2a*, *Tuba1a*, and *Actb*). The impact of 6-month morphine withdrawal on the changes in the proteomic profiles was higher in the hippocampus—14 proteins, only three proteins were detected in the forebrain cortex. Gene Ontology (GO) enrichment analysis of differentially expressed hippocampal proteins revealed the most enriched terms related to metabolic changes, cytoskeleton organization and response to oxidative stress. There is increasing evidence that energy metabolism plays an important role in opioid addiction. However, the way how morphine treatment and withdrawal alter energy metabolism is not fully understood. Our results indicate that the rat hippocampus is more susceptible to changes in proteome and phosphoproteome profiles induced by 6-month morphine withdrawal than is the forebrain cortex.

## 1. Introduction

Morphine is still considered a frequently used opioid in the treatment of moderate to severe pain. However, repetitive clinical use has many negative side effects [1,2]. The analgesic effect is caused by the activation of the opioid receptors (ORs). It has a high affinity for µ-OR (MOR) and a lower affinity for κ-OR (KOR) and δ-OR (DOR) [3,4].

During the last years, we published several animal studies related to the consequences of morphine treatment and withdrawal. Bourova et al. [5] described that exposure of rats to increasing doses of morphine (10–50 mg/kg, 10 days) results in significant desensitization of μ-OR- and δ-OR-stimulated G protein response in the rat forebrain cortex. These findings were in agreement with the data published previously [6,7,8,9,10,11]. Ujcikova et al. [12] detected specific increased level of adenylyl cyclase I (8-fold) and adenylyl cyclase II (2.5-fold) in rat brain cortical plasma membrane samples after 10-day morphine treatment which returned to control level after 20 days of morphine withdrawal. There was no change in the expression level of other adenylyl cyclase isoforms (III-IX). Quantitative immunoblot analysis indicated the unchanged level of G protein α and β subunits: Gα_i1_/Gα_i2_, Gα_i3_, Gα_o_, Gα_q_/Gα_11_, Gα_s_, Gα_z_, and Gβ. The same applied to Na, K-ATPase, and caveolin-1 [12,13].

We applied 2D electrophoretic proteomic approach accompanied by label-free quantification to analyze the altered proteins in the rat brain after 10-day morphine administration followed by protracted drug abstinence [14,15,16,17]. The identified proteins were mainly involved in the change of energy metabolism, regulation of the cytoskeleton, signal transduction, oxidative stress pathways, and apoptotic pathways. Similar to our results, a number of studies indicated that chronic morphine treatment causes significant changes in the expression level of metabolic enzymes, cytoskeletal proteins, and apoptosis-related proteins [18,19,20,21,22,23,24,25,26,27]. These data may support the idea that long-term morphine treatment dysregulates brain energy homeostasis, increases the degree of neuroplasticity, and causes the state of brain cell discomfort [14,15].

Interestingly, we obtained the opposite results in the rat brain cortex and hippocampus. The number of altered proteins was decreased in the cortical samples and increased in the hippocampus after 20 days of morphine withdrawal [16]. Proteomic and phosphoproteomic comparison of the four rat brain parts (cortex, hippocampus, striatum, and cerebellum) isolated from animals after 3 months of drug abstinence demonstrated that withdrawal symptoms may last for weeks or even longer [17]. In the present study, we extended the withdrawal period from 3 to 6 months and applied gel-based proteomics to analyze the alterations in both protein expression and protein phosphorylation in the rat hippocampus and forebrain cortex. This could be a valuable addition to our previous study in which label-free quantification (MaxLFQ) was used to identify changes in the proteomic profiles of these brain parts [28]. Although shotgun proteomics may generate a large list of proteins, it does not bring information about protein isoforms and post-translational modifications. For this purpose, 2D gel electrophoresis is the only currently available proteomic technique [29].

## 2. Materials and Methods

### 2.1. Chemicals

Chemicals for 2D electrophoresis (Immobiline DryStrips, pH 3–11 NL, 13 cm) were obtained from Cytiva (Marlborough, MA, USA), Invitrogen^TM^ (Pro-Q^®^ Diamond Phosphoprotein Gel Stain and Gel Destaining Solution) and Sigma-Aldrich (St. Louis, MO, USA) as described by Ujcikova et al. [17].

### 2.2. Morphine Administration and Drug Withdrawal of Male Wistar Rats

Rats (8 weeks of age) were exposed to increasing doses of morphine (dissolved in 0.9% NaCl) for 10 days (10–50 mg/kg) in parallel with corresponding control animals according to our previously established protocols [5,12,14,15,16,17,28] approved by the Ministry of Education, Youth and Sports of the Czech Republic (license number MSMT-1479/2019–6). Male Wistar rats were housed in the group of 3 per plastic cage on a 12/12 light/dark cycle. Food and water were available ad libitum. Described procedures were performed in an agreement with national and institutional guidelines for the care and use of animals in laboratory research.

### 2.3. Preparation of Samples

According to our experience and considering the sufficient amount of biological material needed for analyses, we used 9 animals of each testing group for the isolation of the brain cortex, hippocampus, striatum and cerebellum. The tissue from 3 randomly selected animals within the same group was pooled into one sample to obtain three equal amounts of brain tissues.

Finally, we obtained six pooled hippocampal (M1, M2, M3, C1, C2, C3), six pooled cortical (M1, M2, M3, C1, C2, C3), six pooled striatal (M1, M2, M3, C1, C2, C3), and six pooled cerebellar (M1, M2, M3, C1, C2, C3) samples which were homogenized as described by Ujcikova et al. [17] and Drastichova et al. [28]. Protein concentration was determined by Lowry method.

### 2.4. Detection of Phosphoproteins by Pro-Q^®^ Diamond Staining

Isoelectric focusing of samples containing 1 mg protein was performed according to our previously established scheme: 150 V for 5 h, 500 V for 1 h, 3500 V for 12 h, and 500 V for 3 h [15,16,17]. SDS-PAGE was followed by gel fixation in 250 mL of 50% methanol/10% acetic acid for 30 min and overnight with gentle agitation [30]. After three times washing in ultrapure water for 10 min, the gels were incubated in 160 mL of Pro-Q^®^ Diamond stain solution for 120 min in the dark. Pro-Q^®^ Diamond gel destaining solution was used three times for 30 min in the dark [17].

### 2.5. Scanning of Phosphorylated Proteins

2D gels were scanned by using Amersham Typhoon Biomolecular Imager (GE Healthcare). Laser excitation wavelength: 532 nm (green), emission filter wavelength: Cy3, 560–580 nm. Scan speed: normal, pixel size: 100 µm, voltage of the photo-multipliertube (PMT): 750 V.

### 2.6. Staining by Colloidal Coomassie Blue (CBB)

CBB staining was used for detection of protein spots on 2D gels and subsequent mass spectrometric analysis as described in [14,15,16].

### 2.7. Statistical Analysis

The PDQuest^TM^ software (Bio-Rad, version 7.3.1, Hercules, CA, USA) was used for evaluation of 2D gels. Protein spots were then checked manually. Relative abundances of protein spots showing significant quantitative differences at least 1.4-fold (*p* ≤ 0.05) were selected for mass spectrometric analysis. *p*-values were calculated by using unpaired Student’s *t*-test and GraphPad*Prism* 8.3.0. Gene Ontology (GO) enrichment analysis of proteomic and phosphoproteomic profiles for rat brain hippocampus and cortex was performed using ShinyGO v0.74 tool in 20 October 2021 (bioinformatics.sdstate.edu/go, accessed on 20 October 2021); the *p*-value cut-off (FDR) was set to 0.05 for biological processes.

### 2.8. nLC-MS/MS

Protein spots were excised from the polyacrylamide gels and then analyzed as described in [17,31,32]. Briefly, after purification with STAGE-TIPs, peptide separation was achieved using a nano-LC device (Proxeon, Odense, Denmark) coupled to a maXis Q-TOF (quadrupole-time of flight) mass spectrometer with ultra-high resolution (Bruker Daltonics, Bremen, Germany). Appropriate software was used (HyStar 3.2 and MicroTOF control Version 3.0., ProteinScape 3.0 and DataAnalysis 4.0 (Bruker Daltonics, Billerica, MA, USA)) for data analysis. Only significant hits (MASCOT score ≥80 for proteins; ≥30 for peptides) were accepted. Proteins were identified by correlating tandem mass spectra with the UniProt/Swiss-Prot database (taxonomy = *Rattus norvegicus*). The MASCOT online search engine (http://www.matrixscience.com) was used. All nLC-MS/MS analyses were performed in duplicates (two samples per spot).

## 3. Results

### 3.1. Proteomic Analysis of the Rat Hippocampus Isolated from Animals after 6 Months of Morphine Withdrawal

#### 3.1.1. Pro-Q^®^ Diamond Staining and Colloidal Coomassie Blue Staining of 2D Gels

**Figure 1:**
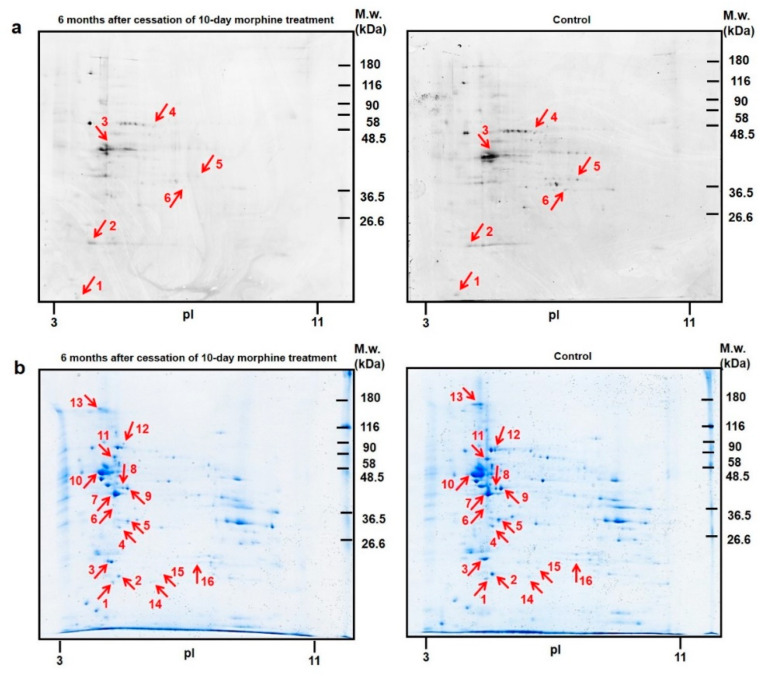
Representative 2D gel maps of phosphorylated proteins (**a**) and total protein profiles (**b**) in the rat hippocampus isolated from animals after 6 months of morphine withdrawal. Red arrows and numbers show the significantly altered protein spots.

Pro-Q^®^ Diamond staining and PDQuest analysis detected 82 protein spots in the rat hippocampus. Among these, six were significantly altered (*p* ≤ 0.05). nLC-MS/MS analysis identified three upregulated proteins (calmodulin-1 ↑2.3-fold, spot **1**; 14-3-3 protein zeta/delta ↑2.4-fold, spot **2**; tubulin alpha-1B chain ↑3.2-fold, spot **3**) and three downregulated proteins (stress-induced-phosphoprotein 1 ↓3.5-fold, spot **4**; phosphoglycerate kinase 1 ↓2.2-fold, spot **5**; fructose-bisphosphate aldolase A ↓2.7-fold, spot **6**), Figure 1a, Table 1a.

Colloidal Coomassie Blue staining (CBB) revealed 111 protein spots, 16 spots were found to differ significantly. Eleven altered proteins were downregulated (peroxiredoxin-2 ↓3.1-fold, spot **1**; phosphatidylethanolamine-binding protein 1 ↓2.6-fold, spot **2**; ubiquitin carboxyl-terminal hydrolase isozyme L1 ↓2.0-fold, spot **3**; beta-soluble NSF attachment protein ↓2.4-fold, spot **4**; guanine nucleotide-binding protein G(I)/G(S)/G(T) subunit beta-1 ↓2.4-fold, spot **5**; endophilin-A1 ↓7.8-fold, spot **6**; actin, cytoplasmic 1 ↓1.6-fold, spot **7**; creatine kinase B-type ↓1.6-fold and ↓2.3-fold, spot **8** and spot **9**; tubulin beta chain ↓1.9-fold and ↓6.1-fold, spot **10** and spot **13**; alpha internexin ↓2.4-fold, spot **11**; stress-70 protein, mitochondrial ↓3.1-fold, spot **12**), and three proteins were upregulated (ATP synthase subunit d, mitochondrial ↑6.7-fold, spot **14**; Parkinson disease protein 7 homolog ↑2.0-fold, spot **15**; triosephosphate isomerase ↑1.9-fold, spot **16**), Figure 1b, Table 1b.

**Figure 2:**
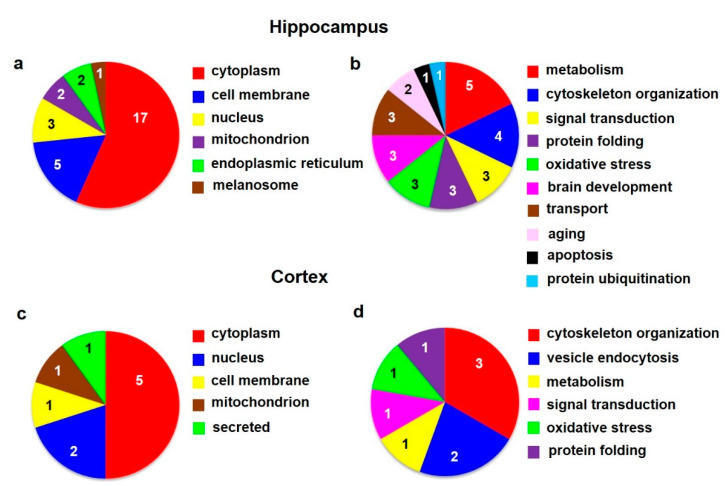
Subcellular localization (**a**,**c**) and function (**b**,**d**) of altered proteins identified in the rat hippocampus (**upper panels**) and cortex (**lower panels**) isolated from animals after 6 months of morphine withdrawal; according to the current annotations (https://www.uniprot.org) in the UniProt database.

According to the current annotations (https://www.uniprot.org, accessed on 20 October 2021) in the UniProt database, the identified hippocampal proteins were involved in *metabolism* (5), *cytoskeleton organization* (4), *signal transduction* (3), *protein folding* (3), *response to oxidative stress* (3), *brain development* (3), *transport* (3), *aging* (2), *apoptosis* (1), and *protein ubiquitination* (1) (Figure 2b; Table 2a,b).

#### 3.1.2. GO Enrichment Analysis of Altered Hippocampal Proteins

**Figure 3:**
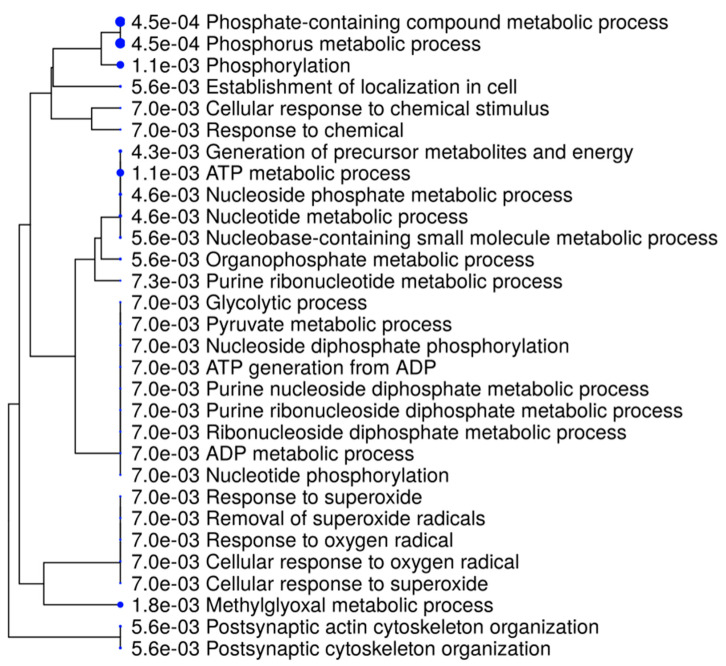
Hierarchical clustering tree summarizing the top 30 most significantly enriched GO terms that were identified in rat hippocampus. The analyzed dataset was consisted of 20 significantly differentially expressed hippocampal proteins.

GO enrichment analysis of 20 significantly differentially expressed hippocampal proteins was carried out using the ShinyGO v0.74 tool (bioinformatics.sdstate.edu/go). The top thirty most significantly enriched GO terms for biological processes were summarized in hierarchical clustering tree, see Figure 3. The most enriched GO terms were related to *metabolic changes*: phosphorus metabolic process, phosphate-containing compound metabolic process, ATP metabolic process, methylglyoxal metabolic process, generation of precursor metabolites and energy, nucleoside phosphate metabolic process, nucleotide metabolic process; *cytoskeleton organization*: postsynaptic cytoskeleton organization, establishment of localization in cell, postsynaptic actin cytoskeleton organization, and *oxidative stress*: removal of superoxide radicals, response to superoxide, response to oxygen radical. The detailed data of these GO enriched terms are listed in Table 3, including enrichment FDR values and gene names of altered proteins associated with GO terms.

### 3.2. Proteomic Analysis of the Rat Forebrain Cortex Isolated from Animals after 6 Months of Morphine Withdrawal

#### 3.2.1. Pro-Q^®^ Diamond Staining and Colloidal Coomassie Blue Staining of 2D Gels

**Figure 4:**
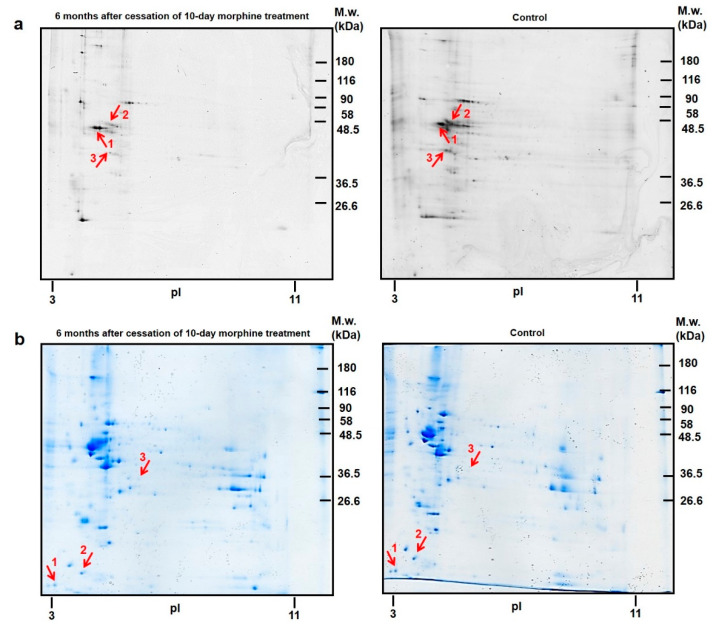
Representative 2D gel maps of phosphorylated proteins (**a**) and total protein profiles (**b**) in the rat forebrain cortex isolated from animals after 6 months of morphine withdrawal. Red arrows and numbers show the significantly altered protein spots.

The 83 phosphorylated protein spots were detected in the rat brain cortex, only three were significantly downregulated: tubulin beta-2A chain ↓2.4-fold, spot **1**; tubulin alpha-1A chain ↓3.7-fold, spot **2** and actin, cytoplasmic 1 ↓2.7-fold, spot **3**; Figure 4a, Table 1c.

CBB staining revealed 87 protein spots in cortical 2D gels, only three of these were significantly altered. nLC-MS/MS analysis identified two proteins with decreased level (calmodulin 1 ↓2.0-fold, spot **1**; alpha-synuclein ↓1.4-fold, spot **2**) and one upregulated protein: mitochondrial isocitrate dehydrogenase [NAD] subunit alpha ↑2.4-fold, spot **3**; Figure 4b, Table 1d.

According to the current annotations (https://www.uniprot.org) in the UniProt database, the identified cortical proteins were functionally related to *cytoskeleton organization* (3), *vesicle endocytosis* (2), *metabolism* (1), *signal transduction* (1), *response to oxidative stress* (1), and *protein folding* (1) (Figure 2d; Table 2c,d).

#### 3.2.2. GO Enrichment Analysis of Altered Cortical Proteins

**Figure 5:**
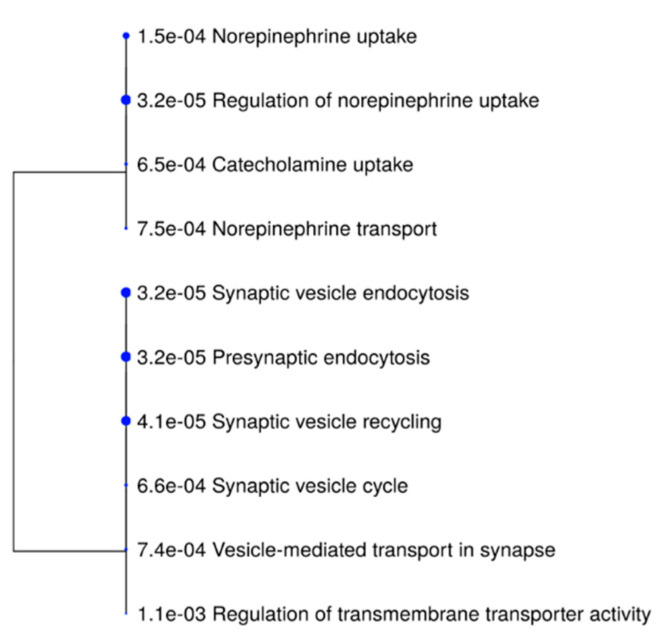
Hierarchical clustering tree summarizing the top 10 most significantly enriched GO terms that were identified in rat forebrain cortex. The analyzed dataset was consisted of 6 significantly differentially expressed cortical proteins.

GO enrichment analysis of six significantly differentially expressed cortical proteins (Table 1c,d) was carried out using the ShinyGO v0.74 tool (bioinformatics.sdstate.edu/go). The top ten most significantly enriched GO terms for biological processes were summarized in hierarchical clustering tree (Figure 5). The most enriched GO terms were related to *vesicle endocytosis*: presynaptic endocytosis, synaptic vesicle endocytosis, synaptic vesicle recycling, synaptic vesicle cycle, vesicle-mediated transport in synapse, and *regulation of catecholamine uptake*. The detailed data of these GO enriched terms are listed in Table 4, including enrichment FDR values and gene names of altered proteins associated with GO terms.

### 3.3. Proteomic Analysis of the Rat Striatum and Cerebellum Isolated from Animals after 6 Months of Morphine Withdrawal

#### 3.3.1. Colloidal Coomassie Blue Protein Staining of Striatal 2D Gels

**Figure 6:**
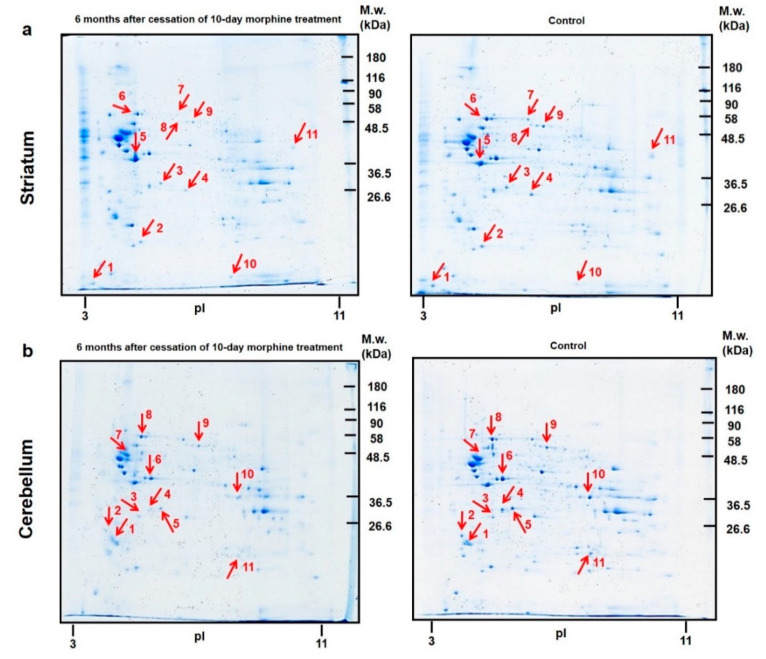
Representative 2D gel maps of total protein profiles in the rat striatum (**a**, **upper panels**) and cerebellum (**b**, **lower panels**) isolated from animals after 6 months of morphine withdrawal. Red arrows and numbers show the significantly altered protein spots.

CBB-stained 2D gels revealed 152 protein spots in the rat striatum, ten proteins with significantly changed expression level were identified by nLC-MS/MS analysis. Among these, seven proteins were downregulated: calmodulin-1 ↓2.3-fold, spot **1**; l-lactate dehydrogenase B chain ↓1.4-fold, spot **3**; malate dehydrogenase, cytoplasmic ↓1.8-fold, spot **4**; albumin ↓2.4-fold, spot **7**; dihydropyrimidinase-related protein 2 ↓1.7-fold and ↓2.0-fold, spot **8** and spot **9**; nucleoside diphosphate kinase B ↓2.0-fold, spot **10**, and elongation factor 1-alpha ↓3.1-fold, spot **11**. Only three proteins were upregulated: peroxiredoxin-2 ↑1.8-fold, spot **2**; actin, cytoplasmic 1 ↑3.3-fold, spot **5** and heat shock cognate 71 kDa protein ↑2.5-fold, spot **6**; Figure 6a, Table 5a.

**Figure 7:**
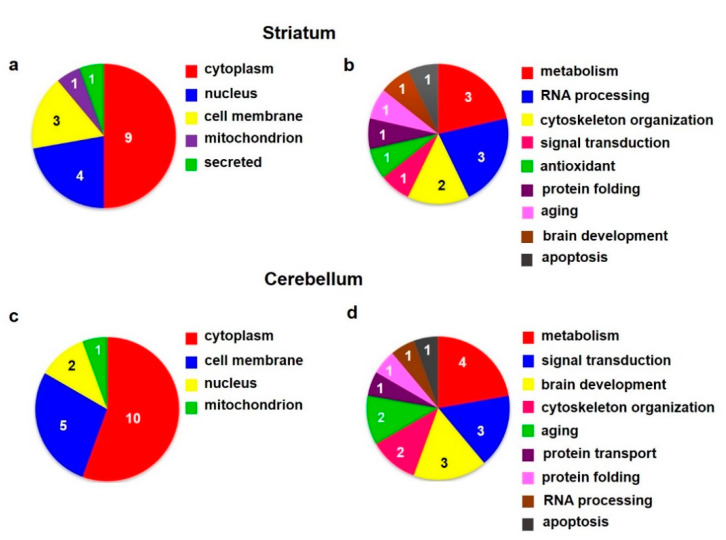
Subcellular localization (**a**,**c**) and function (**b**,**d**) of altered proteins identified in the rat striatum (**upper panels**) and cerebellum (**lower panels**) isolated from animals after 6 months of morphine withdrawal; according to the current annotations (https://www.uniprot.org) in the UniProt database.

According to the current annotations (https://www.uniprot.org) in the UniProt database, the altered striatal proteins were found to be functionally related to *metabolism* (3), *RNA processing* (3), *cytoskeleton organization* (2), *signal transduction* (1), *response to oxidative stress* (1), *protein folding* (1), *aging* (1), *brain development* (1), and *apoptosis* (1) (Figure 7b; Table 6a).

#### 3.3.2. Colloidal Coomassie Blue Protein Staining of Cerebellar 2D Gels

The total number of 122 protein spots were detected in the rat cerebellum, eleven proteins were significantly downregulated: 14-3-3 protein gamma ↓3.8-fold, spot **1**; 14-3-3 protein epsilon ↓2.6-fold, spot **2**; beta-soluble NSF attachment protein ↓1.6-fold, spot **3**; guanine nucleotide-binding protein G(I)/G(S)/G(T) subunit beta-1 ↓4.4-fold, spot **4**; l-lactate dehydrogenase B chain ↓1.9-fold, spot **5**; creatine kinase ↓1.5-fold, spot **6**; tubulin alpha-1B chain ↓2.1-fold, spot **7**; heat shock cognate 71 kDa protein ↓2.1-fold, spot **8**; dihydropyrimidinase-related protein 2 ↓1.8-fold, spot **9**; fructose-bisphosphate aldolase C ↓3.0-fold, spot **10** and triosephosphate isomerase ↓2.2-fold, spot **11**. Upregulation was not detected (Figure 6b; Table 5b).

According to the current annotations (https://www.uniprot.org) in the UniProt database, the identified cerebellar proteins with changed expression level were involved in *metabolism* (4), *signal transduction* (3), *brain development* (3), *cytoskeleton organization* (2), *aging* (2), *protein transport* (1), *protein folding* (1), *RNA processing* (1), and *apoptosis* (1) (Figure 7d; Table 6b).

## 4. Discussion

During the last years, we applied 2D electrophoresis and label-free quantification to find the significant alterations in protein expression in the rat brain cortex and hippocampus after chronic morphine treatment (10–50 mg/kg, 10 days) followed by different withdrawal periods (3 weeks, 3 months, 6 months) [15,16,17,28]. The aim of this work was to analyze the spectrum of altered proteins in selected rat brain regions after 6-month morphine withdrawal. Our study could have two main limitations. First, some animals could be lost during morphine administration and withdrawal. For that reason, we had three extra animals in each testing group. Second, proteomic analyses require a large amount of tissue. For this purpose, we selected the stated brain regions (cortex, hippocampus, striatum, and cerebellum). This selection provided us with a relatively large amount of biological material.

2D-DIGE analysis of the rat hippocampus showed that 10-day morphine administration results in a significant change of six proteins functionally related to metabolism, cytoskeleton organization, neuronal plasticity, apoptosis, and oxidative stress. Interestingly, the number of differentially regulated proteins was increased to 13 after 3 weeks of drug abstinence. Moreover, the level of α-synuclein (*Snca*), β-synuclein (*Sncb*), α-enolase (*Eno1*), and glyceraldehyde-3-phosphate dehydrogenase (GAPDH) persisted altered for 3 weeks since the withdrawal of morphine [16]. Ten proteins were identified in hippocampal 2D CBB-stained gels 3 months after cessation of 10-day morphine treatment; 14 proteins were significantly hypophosphorylated [17]. Among these, several glycolytic enzymes, such as GAPDH, *Eno1*, phosphoglycerate mutase 1 (*Pgam1*), triosephosphate isomerase (*Tpi1*) and fructose-bisphosphate aldolase A (*Aldoa*) were decreased. In this work, the impact of 6-month morphine withdrawal on the change of total protein composition was higher-14 proteins were identified (Figure 1b; Table 1b). On the other hand, the number of dysregulated phosphoproteins was reduced from 14 to 6 (Figure 1a; Table 1a). The amount of glycolytic enzymes with significantly changed expression level was not so eminent. Pro-Q^®^ Diamond staining revealed downregulation of *Aldoa* and an increased level of *Tpi1* was detected by CBB protein staining.

Previous studies of other authors have shown that enzymes involved in cellular metabolisms, such as glycolysis and Krebs cycle were altered in opioid-abusing patients and animal models [33,34]. Among the glycolytic enzymes, GAPDH is of particular interest. Its post-translational modifications may contribute to numerous cellular functions, including intracellular transport, cytoskeleton plasticity, heme chaperoning, transcription, and apoptosis [18,35,36,37,38,39,40]. However, its role in apoptosis is not clear. Some studies describe its proapoptotic function, others a protective role [41]. One of the typical features of GAPDH is its use as a loading marker in hundreds of studies. Notably, it was shown that the quantity of GAPDH can vary under stressful conditions [42].

Decreased level of superoxide dismutase [Cu-Zn] (*Sod1*) was detected in our hippocampal samples after 3 weeks of drug abstinence [16]. In this work, 6-month morphine withdrawal revealed downregulation of peroxiredoxin-2 (*Prdx2*) and upregulation of Parkinson disease protein 7 homolog (*Park7*), see Table 1b. *Park 7* is involved in the protection against oxidative stress [43]. Due to its protective role, *Park 7* represents an ideal possible therapeutic target for Parkinson’s disease (PD) and neurodegeneration [44]. We may hypothesize that stress-related pathways become activated during opioid withdrawal and can persist for several months after cessation of morphine administration.

Morphine could participate in the development of oxidative stress by promoting the formation of free radicals or reducing the activity of the antioxidant defense system which maintain redox homeostasis. Both these ways of action can be possibly combined [45]. Among the most important molecules playing a crucial role in cell protection against oxidative damage belong enzymes such as superoxide dismutase, glutathione peroxidase, catalase, and tripeptide glutathione [46]. The activity of antioxidant enzymes is closely associated with the production of reactive oxygen species (ROS) and reactive nitrogen species (RNS) that may lead to oxidative damage of DNA, lipids and proteins [47,48]. Results obtained by Motaghinejad et al. [49] showed that subcutaneous injection of morphine to rats significantly increased lipid peroxidation and decreased the activities of superoxide dismutase and glutathione peroxidase. Abdel-Zaher et al. [50] reported that glutamate levels and lipid peroxide malondialdehyde levels were significantly increased in the brain of morphine-treated mice. The impact of morphine on cellular redox balance may depend on multiple factors, such as species, age and sex of an organism, type of tissue, dosage, and length of usage [51].

The number of altered phosphorylated cytoskeletal proteins in the rat hippocampus was decreased from four (hypophosphorylation of F-actin-capping subunit beta (*Capzb*); actin, cytoplasmic 2 (*Actg1*); glial fibrillary acidic protein (GFAP) and tubulin alpha-1A chain (*Tuba1a*)) to one hyperphosphorylated tubulin alpha-1B chain (*Tuba1b*) when compared the period of abstinence 3 and 6 months. The change in protein expression was almost similar after 3 or 6 months of drug withdrawal and resulted in a decreased level of tubulin beta-4B chain (*Tubb4b*), tubulin polymerization promoting protein (*Tppp*), actin, cytoplasmic 1 (*Actb*), tubulin beta chain (*Tubb4b*), and alpha-internexin (*Ina*). These findings suggest that protracted morphine abstinence may cause long-term homeostatic changes in hippocampal plasticity [52]. However, according to our unpublished behavioral studies, we did not find significant differences between morphine-withdrawn and control animals. We may speculate that proteomic changes in the rat hippocampus after 6 months of morphine withdrawal do not require alterations in a certain behavior or functional state.

Twenty-eight significantly altered proteins were detected in the rat brain cortex after treatment with morphine for 10 days, this amount was reduced to 14 proteins after 3 weeks of abstinence [15], to 10 proteins after 3-month morphine withdrawal [17] and to only three proteins after 6-month drug abstinence. Chronic morphine treatment resulted in the decreased level of several glycolytic enzymes, such as *Tpi1*, *Pgam1*, GAPDH, *Aldoa*, pyruvate kinase PKM (*Pkm*), and phosphoglycerate kinase 1 (*Pgk1*). The expression level of *Pkm*, *Pgk1* and GAPDH persisted decreased for 3 months of drug withdrawal and was not altered after 6 months of abstinence. It would be useful to confirm whether proteomics-indicated alterations in enzyme levels reflect changes in their activity. The change in protein expression does not necessarily mean the change in functional activity, as described by Bodzon-Kulakowska et al. [53] and Antolak et al. [26]. We assume that simultaneous alterations in both features may represent new insight into brain energy homeostasis.

Increasing evidence suggests that the striatum and cerebellum participate in drug addiction [54,55,56,57,58]. As a complement to proteomic analyses of rat hippocampus and cortex, we also performed the screening of protein alterations in the rat striatum and cerebellum after 6-month morphine withdrawal. Interestingly, the number of altered proteins was increased in both the striatum and cerebellum after 6-month drug withdrawal in comparison with the effect of 3-month morphine abstinence. In the striatum, the number of differentially expressed proteins was increased from 7 to 10 while in the cerebellum from 4 to 11. The majority of changes were related to metabolic alterations (L-lactate dehydrogenase B chain (*Ldhb*), malate dehydrogenase (*Ldh1*), creatine kinase B-type (*Ckb*), fructose-bisphosphate aldolase C (*Aldoc*), and *Tpi1*) (Figure 6; Table 6a,b). Taken together, our results suggest that protracted morphine withdrawal causes significant proteomic changes in the energy metabolism of different rat brain parts. We assume that deeper metabolic investigation into the brain structures may reveal numerous differences in glucose metabolism, the tricarboxylic acid cycle (TCA) and fatty acid metabolism. In addition, we may expect changes in metabolites related to antioxidant and nucleotide pathways. However, detailed studies are missing. 

## 5. Conclusions

Our data show that the rat hippocampus is more affected than the forebrain cortex in both protein phosphorylation and protein expression by 6-month morphine withdrawal. Gene Ontology (GO) enrichment analysis for 20 up- and downregulated proteins in the hippocampus revealed that the most enriched GO terms were associated with alterations in energy metabolism, cytoskeleton organization, and oxidative stress response. Our previous proteomic studies indicated that 10-day morphine administration results in significant alterations related to energy metabolism. Moreover, these changes persisted several weeks/months after the cessation of 10-day morphine treatment. We hypothesize that alterations in energy metabolism may be one of the functional consequences of the impaired antioxidant defense system. However, these questions need further investigation.

## Figures and Tables

**Table 1:** nLC-MS/MS analysis of significantly altered protein spots identified in the rat hippocampus (**a**,**b**) and cortex (**c**,**d**) isolated from animals after 6 months of morphine withdrawal.

Spot	Accession	Gene	Protein Name	Mascot	Matched	SC ^a^	MW ^b^	pI ^c^	Change	*p* Value
	Number			Score	Peptides	[%]	(kDa)		(Fold)	
***(a) HIPPOCAMPUS Pro-Q^®^ Diamond staining***										
1	P0DP29	Calm1	Calmodulin-1	2299.5	28	93.3	16.8	3.9	↑2.3	0.0316
2	P63102	Ywhaz	14-3-3 protein zeta/delta	1654.2	25	74.7	27.8	4.6	↑2.4	0.0014
3	Q6P9V9	Tuba1b	Tubulin alpha-1B chain	1866.7	27	57.9	50.1	4.8	↑3.2	0.0424
4	O35814	Stip1	Stress-induced phosphoprotein 1	1728.2	32	39.4	62.5	6.4	↓3.5	0.0309
5	P16617	Pgk1	Phosphoglycerate kinase 1	2026.8	36	57.8	44.5	9.0	↓2.2	0.0195
6	P05065	Aldoa	Fructose-bisphosphate aldolase A	2243.8	35	80.2	39.3	9.2	↓2.7	0.0084
***(b) HIPPOCAMPUS Colloidal Coommassie Blue staining***										
1	P35704	Prdx2	Peroxiredoxin-2	429.6	8	33.8	21.8	5.2	↓3.1	0.0036
2	P31044	Pebp1	Phosphatidylethanolamine-binding	988.5	13	62.6	20.8	5.4	↓2.6	0.0004
			protein 1							
3	Q00981	Uchl1	Ubiquitin carboxyl-terminal hydrolase	1462.4	24	59.6	24.8	5.0	↓2.0	0.0394
			isozyme L1							
4	F8WFM2	Napb	Beta-soluble NSF attachment protein	1276.3	20	55.4	33.5	5.2	↓2.4	0.0015
5	P54311	Gnb1	Guanine nucleotide-binding protein	728.3	12	33.2	37.4	5.6	↓2.4	0.0045
			G(I)/G(S)/G(T) subunit beta-1							
6	O35179	Sh3gl2	Endophilin-A1	1368.9	28	34.4	39.9	5.1	↓7.8	0.0363
7	P60711	Actb	Actin, cytoplasmic 1	2492.6	38	62.7	41.7	5.2	↓1.8	0.0293
8	P07335	Ckb	Creatine kinase B-type	2008.6	27	57	42.7	5.3	↓1.6	0.0402
9	P07335	Ckb	Creatine kinase B-type	2337.6	34	65.4	42.7	5.3	↓2.3	0.0026
10	G3V7C6	Tubb4b	Tubulin beta chain	2983.9	37	49.8	61.1	4.6	↓1.9	0.0358
11	P23565	Ina	Alpha-internexin	2688.1	41	75.6	56.1	5.1	↓2.4	0.0117
12	F1M953	Hspa9	Stress-70 protein, mitochondrial	2772.9	43	41.7	73.7	5.8	↓3.1	0.0127
13	G3V7C6	Tubb4b	Tubulin beta chain	1809.5	23	40.5	61.1	4.6	↓6.1	0.0175
14	P31399	Atp5pd	ATP synthase subunit d, mitochondrial	1680.6	23	67.7	18.8	6.2	↑6.7	0.0011
15	O88767	Park7	Parkinson disease protein 7 homolog	1106.3	15	70.4	20	6.4	↑2.0	0.0324
16	P48500	Tpi1	Triosephosphate isomerase	829.1	12	53	26.8	7.7	↑1.9	0.0348
***(c) CORTEX Pro-Q^®^ Diamond staining***										
1	P85108	Tubb2a	Tubulin beta-2A chain	3071.4	37	73	49.9	4.6	↓2.4	0.0103
2	P68370	Tuba1a	Tubulin alpha-1A chain	1538.3	21	47	50.1	4.8	↓3.7	0.0406
3	P60711	Actb	Actin, cytoplasmic 1	1532.3	23	50.9	41.7	5.2	↓2.7	0.0284
***(d) CORTEX Colloidal Coommassie Blue staining***										
1	P0DP29	Calm1	Calmodulin-1	1670.9	18	74.5	16.8	3.9	↓2.0	0.0478
2	P37377	Snca	Alpha-synuclein	1287.2	16	80	14.5	4.6	↓1.4	0.0114
3	Q99NA5	Idh3a	Isocitrate dehydrogenase [NAD] subunit	830.1	9	28	39.6	6.5	↑2.4	0.0252
			alpha, mitochondrial							

^a^ sequence coverage, ^b^ theoretical molecular weight, ^c^ theoretical isoelectric point.

**Table 2:** Subcellular localization and function of altered proteins identified in the rat hippocampus (**a**,**b**) and cortex (**c**,**d**) isolated from animals after 6 months of morphine withdrawal; according to the current annotations (https://www.uniprot.org) in the UniProt database.

Spot	Accession	Protein Name	Change	Subcellular Localization	GO-Molecular Functions, Biological Processes
	Number		(Fold)		
***(a) HIPPOCAMPUS Pro-Q^®^ Diamond staining***					
1	P0DP29	Calmodulin-1	↑2.3	Cytoplasm, cytoskeleton	Calcium-mediated signaling, activation
					of adenylate cyclase activity, regulation
					of cytokinesis
2	P63102	14-3-3 protein zeta/delta	↑2.4	Cytoplasm, melanosome	Signal transducing adaptor protein
3	Q6P9V9	Tubulin alpha-1B chain	↑3.2	Cytoplasm, cytoskeleton	Cell shape and movement
4	O35814	Stress-induced phosphoprotein 1	↓3.5	Cytoplasm, nucleus	Chaperone binding
5	P16617	Phosphoglycerate kinase 1	↓2.2	Cytoplasm	Energy metabolism (glycolysis)
6	P05065	Fructose-bisphosphate aldolase A	↓2.7	Cytoplasm	Energy metabolism (glycolysis)
***(b) HIPPOCAMPUS Colloidal Coommassie Blue staining***					
1	P35704	Peroxiredoxin-2	↓3.1	Cytoplasm	Antioxidant, response to oxidative stress
2	P31044	Phosphatidylethanolamine-binding	↓2.6	Cytoplasm, cell membrane	Hippocampus development, aging, response
		protein 1			to oxidative stress, MAPK cascade
3	Q00981	Ubiquitin carboxyl-terminal hydrolase	↓2.0	Cytoplasm, endoplasmic reticulum	Protein ubiquitination, axonogenesis
		isozyme L1			
4	F8WFM2	Beta-soluble NSF attachment protein	↓2.4	Cell membrane	ER-Golgi transport, protein transport
5	P54311	Guanine nucleotide-binding protein	↓2.4	Cell membrane, cytoplasm	Signal transducer
		G(I)/G(S)/G(T) subunit beta-1			
6	O35179	Endophilin-A1	↓7.8	Cytoplasm, endosome, cell membrane	Endocytosis, regulation of receptor internalization
7	P60711	Actin, cytoplasmic 1	↓1.8	Cytoplasm, cytoskeleton, nucleus	Cell shape and movement
8,9	P07335	Creatine kinase B-type	↓1.6, ↓2.3	Cytoplasm	Brain development, creatine metabolism
10,13	G3V7C6	Tubulin beta chain	↓1.9, ↓6.1	Cytoplasm, cytoskeleton	Cell shape and movement
11	P23565	Alpha-internexin	↓2.4	Cytoplasm, cytoskeleton	Cytoskeleton organization, developmental protein
12	F1M953	Stress-70 protein, mitochondrial	↓3.1	Mitochondrion	Chaperone
14	P31399	ATP synthase subunit d, mitochondrial	↑6.7	Mitochondrion	ATP metabolic process, hydrogen ion transport
15	O88767	Parkinson disease protein 7 homolog	↑2.0	Cell membrane, cytoplasm, nucleus,	Chaperone, aging, inflammatory response, stress
				endoplasmic reticulum	response, negative regulation of apoptosis
16	P48500	Triosephosphate isomerase	↑1.9	Cytoplasm	Energy metabolism (glycolysis)
***(c) CORTEX Pro-Q^®^ Diamond staining***					
1	P85108	Tubulin beta-2A chain	↓2.4	Cytoplasm, cytoskeleton	Cell shape and movement
2	P68370	Tubulin alpha-1A chain	↓3.7	Cytoplasm, cytoskeleton	Cell shape and movement
3	P60711	Actin, cytoplasmic 1	↓2.7	Cytoplasm, cytoskeleton, nucleus	Cell shape and movement
***(d) CORTEX Colloidal Coommassie Blue staining***					
1	P0DP29	Calmodulin-1	↓2.0	Cytoplasm, cytoskeleton	Calcium-mediated signaling, activation of adenylate cyclase activity, regulation of cytokinesis
2	P37377	Alpha-synuclein	↓1.4	Cytoplasm, cell membrane, nucleus, secreted	Chaperone, response to oxidative stress, regulation of synaptic vesicle trafficking, regulation of neurotransmitter release
3	Q99NA5	Isocitrate dehydrogenase [NAD] subunit alpha, mitochondrial	↑2.4	Mitochondrion	Krebs cycle

**Table 3:** GO enrichment analysis for significantly upregulated and downregulated proteins identified in the rat hippocampus isolated from animals after 6 months of morphine withdrawal; carried out using the ShinyGO v0.74 tool (bioinformatics.sdstate.edu/go). The top thirty most significantly enriched GO terms for biological processes are listed.

Pathways	Enrichment FDR	Pathway	Genes	Name of Genes in List
		Genes	in List	
Phosphorus metabolic process	0.000454130956700965	2813	12	Atp5pd, Aldoa, Uchl1, Prdx2, Calm1, Sh3gl2, Ywhaz, Ckb, Tpi1, Park7, Pgk1, Actb
Phosphate-containing compound metabolic process	0.000454130956700965	2798	12	Atp5pd, Aldoa, Uchl1, Prdx2, Calm1, Sh3gl2, Ywhaz, Ckb, Tpi1, Park7, Pgk1, Actb
Phosphorylation	0.00109269868079895	2072	10	Aldoa, Uchl1, Prdx2, Sh3gl2, Ywhaz, Tpi1, Park7, Pgk1, Actb, Atp5pd
ATP metabolic process	0.00109269868079895	272	5	Atp5pd, Aldoa, Tpi1, Park7, Pgk1
Methylglyoxal metabolic process	0.001813238441941	5	2	Park7, Aldoa
Generation of precursor metabolites and energy	0.00432203205106559	397	5	Aldoa, Tpi1, Park7, Pgk1, Atp5pd
Nucleoside phosphate metabolic process	0.00464706222526931	428	5	Atp5pd, Aldoa, Tpi1, Park7, Pgk1
Nucleotide metabolic process	0.00464706222526931	419	5	Atp5pd, Aldoa, Tpi1, Park7, Pgk1
Postsynaptic cytoskeleton organization	0.00556835599926725	13	2	Ina, Actb
Organophosphate metabolic process	0.00556835599926725	820	6	Atp5pd, Aldoa, Ckb, Tpi1, Park7, Pgk1
Establishment of localization in cell	0.00556835599926725	1630	8	Calm1, Ywhaz, Uchl1, Napb, Park7, Hspa9, Actb, Atp5pd
Nucleobase-containing small molecule metabolic process	0.00556835599926725	473	5	Atp5pd, Aldoa, Tpi1, Park7, Pgk1
Postsynaptic actin cytoskeleton organization	0.00556835599926725	13	2	Ina, Actb
Pyruvate metabolic process	0.00698656482715899	121	3	Aldoa, Tpi1, Pgk1
Glycolytic process	0.00698656482715899	102	3	Aldoa, Tpi1, Pgk1
Nucleoside diphosphate phosphorylation	0.00698656482715899	120	3	Aldoa, Tpi1, Pgk1
ATP generation from ADP	0.00698656482715899	103	3	Aldoa, Tpi1, Pgk1
Purine nucleoside diphosphate metabolic process	0.00698656482715899	113	3	Aldoa, Tpi1, Pgk1
Purine ribonucleoside diphosphate metabolic process	0.00698656482715899	113	3	Aldoa, Tpi1, Pgk1
Ribonucleoside diphosphate metabolic process	0.00698656482715899	116	3	Aldoa, Tpi1, Pgk1
Removal of superoxide radicals	0.00698656482715899	18	2	Prdx2, Park7
Response to chemical	0.00698656482715899	4423	12	Ywhaz, Gnb1, Uchl1, Prdx2, Calm1, Park7, Ina, Stip1, Actb, Tuba1b, Atp5pd, Aldoa
Response to superoxide	0.00698656482715899	21	2	Prdx2, Park7
Response to oxygen radical	0.00698656482715899	21	2	Prdx2, Park7
ADP metabolic process	0.00698656482715899	108	3	Aldoa, Tpi1, Pgk1
Nucleotide phosphorylation	0.00698656482715899	122	3	Aldoa, Tpi1, Pgk1
Cellular response to chemical stimulus	0.00698656482715899	2498	9	Gnb1, Uchl1, Prdx2, Park7, Ina, Stip1, Actb, Tuba1b, Atp5pd
Cellular response to oxygen radical	0.00698656482715899	19	2	Prdx2, Park7
Cellular response to superoxide	0.00698656482715899	19	2	Prdx2, Park7
Purine ribonucleotide metabolic process	0.00726512653650191	327	4	Atp5pd, Aldoa, Tpi1, Pgk1

**Table 4:** GO enrichment analysis for significantly upregulated and downregulated proteins identified in the rat forebrain cortex isolated from animals after 6 months of morphine withdrawal; carried out using the ShinyGO v0.74 tool (bioinformatics.sdstate.edu/go). The top ten most significantly enriched GO terms for biological processes are listed.

Pathways	Enrichment FDR	Pathway	Genes	Name of Genes in List
		Genes	in List	
Presynaptic endocytosis	0.0000317791916695507	41	3	Snca, Actb, Calm1
Synaptic vesicle endocytosis	0.0000317791916695507	41	3	Snca, Actb, Calm1
Regulation of norepinephrine uptake	0.0000317791916695507	2	2	Snca, Actb
Synaptic vesicle recycling	0.0000411603443103439	49	3	Snca, Actb, Calm1
Norepinephrine uptake	0.00014973418795375	6	2	Snca, Actb
Catecholamine uptake	0.000648303871273433	13	2	Snca, Actb
Synaptic vesicle cycle	0.000655582495341169	147	3	Snca, Actb, Calm1
Vesicle-mediated transport in synapse	0.000739942307588717	160	3	Snca, Actb, Calm1
Norepinephrine transport	0.000753222666338899	17	2	Snca, Actb
Regulation of transmembrane transporter activity	0.00113861317223078	199	3	Calm1, Snca, Actb

**Table 5:** nLC-MS/MS analysis of significantly altered protein spots identified in the rat striatum (**a**) and cerebellum (**b**) isolated from animals after 6 months of morphine withdrawal.

Spot	Accession	Gene	Protein Name	Mascot	Matched	SC ^a^	MW ^b^	pI ^c^	Change	*p* Value
	Number			Score	Peptides	[%]	(kDa)		(Fold)	
***(a) STRIATUM Colloidal Coommassie Blue staining***										
1	P0DP29	Calm1	Calmodulin-1	1050.8	15	74.5	16.8	3.9	↓2.3	0.0079
2	P35704	Prdx2	Peroxiredoxin-2	388.2	7	31.3	21.8	5.2	↑1.8	0.0214
3	P42123	Ldhb	L-lactate dehydrogenase B chain	1159.9	20	41.3	36.6	5.6	↓1.4	0.0483
4	O88989	Mdh1	Malate dehydrogenase, cytoplasmic	808.2	14	44.9	36.5	6.2	↓1.8	0.0326
5	P60711	Actb	Actin, cytoplasmic 1	1304.1	24	44.5	41.7	5.2	↑3.3	0.0258
6	P63018	Hspa8	Heat shock cognate 71 kDa protein	1336.6	23	31.6	70.8	5.2	↑2.5	0.0050
7	P02770	Alb	Albumin	651.8	12	18.3	68.7	6.1	↓2.4	0.0115
8	P47942	Dpysl2	Dihydropyrimidinase-related protein 2	1104.7	17	35.5	62.2	5.9	↓1.7	0.0352
9	P47942	Dpysl2	Dihydropyrimidinase-related protein 2	1997.6	31	56.3	62.2	5.9	↓2.0	0.0244
10	P19804	Nme2	Nucleoside diphosphate kinase B	575.2	11	59.2	17.3	7.8	↓2.0	0.0458
11	M0R757	LOC100360413	Elongation factor 1-alpha	548.1	12	21.2	50.1	9.7	↓3.1	0.0030
***(b) CEREBELLUM Colloidal Coommassie Blue staining***										
1	P61983	Ywhag	14-3-3 protein gamma	1065.1	20	49.4	28.3	4.7	↓3.8	0.0451
2	P62260	Ywhae	14-3-3 protein epsilon	1373.3	23	49.8	29.2	4.5	↓2.7	0.0214
3	P85969	Napb	Beta-soluble NSF attachment protein	418.7	9	26.6	33.4	5.2	↓1.6	0.0172
4	P54311	Gnb1	Guanine nucleotide-binding protein	497.7	9	22.9	37.4	5.6	↓4.4	0.0035
			G(I)/G(S)/G(T) subunit beta-1							
5	P42123	Ldhb	L-lactate dehydrogenase B chain	1288.1	22	43.7	36.6	5.6	↓1.9	0.0471
6	P07335	Ckb	Creatine kinase B-type	1531.4	22	53.8	42.7	5.3	↓1.5	0.0246
7	Q6P9V9	Tuba1b	Tubulin alpha-1B chain	1209.4	21	53.7	50.1	4.8	↓2.1	0.0041
8	P63018	Hspa8	Heat shock cognate 71 kDa protein	2437.8	37	45.5	70.8	5.2	↓2.1	0.0072
9	P47942	Dpysl2	Dihydropyrimidinase-related protein 2	1684.2	25	48.8	62.2	5.9	↓1.8	0.0174
10	P09117	Aldoc	Fructose-bisphosphate aldolase C	1956.9	28	59	39.3	6.8	↓3.0	0.0053
11	P48500	Tpi1	Triosephosphate isomerase	859.3	12	50.6	26.8	7.7	↓2.2	0.0200

^a^ sequence coverage, ^b^ theoretical molecular weight, ^c^ theoretical isoelectric point.

**Table 6:** Subcellular localization and function of altered proteins identified in the rat striatum (**a**) and cerebellum (**b**) isolated from animals after 6 months of morphine withdrawal; according to the current annotations (https://www.uniprot.org) in the UniProt database.

Spot	Accession	Protein Name	Change	Subcellular Localization	GO-Molecular Functions, Biological Processes
	Number		(Fold)		
***(a) STRIATUM Colloidal Coommassie Blue staining***					
1	P0DP29	Calmodulin-1	↓2.3	Cytoplasm, cytoskeleton	Calcium-mediated signaling, activation
					of adenylate cyclase activity, regulation
					of cytokinesis
2	P35704	Peroxiredoxin-2	↑1.8	Cytoplasm	Antioxidant, response to oxidative stress
3	P42123	L-lactate dehydrogenase B chain	↓1.4	Cytoplasm, mitochondrion	Pyruvate metabolic process
4	O88989	Malate dehydrogenase, cytoplasmic	↓1.8	Cytoplasm	Krebs cycle
5	P60711	Actin, cytoplasmic 1	↑3.3	Cytoplasm, cytoskeleton, nucleus	Cell shape and movement
6	P63018	Heat shock cognate 71 kDa protein	↑2.5	Cell membrane, cytoplasm, nucleus	Protein folding, RNA processing, aging
7	P02770	Albumin	↓2.4	Secreted	Transporter, apoptosis
8,9	P47942	Dihydropyrimidinase-related protein 2	↓1.7,↓2.0	Cytoplasm, cytoskeleton, cell	Brain development, neurogenesis, cell
				membrane	movement
10	P19804	Nucleoside diphosphate kinase B	↓2.0	Cytoplasm, nucleus	Nucleotide metabolism, transcription
11	M0R757	Elongation factor 1-alpha	↓3.1	Cell membrane, cytoplasm, nucleus	Translation
***(b) CEREBELLUM Colloidal Coommassie Blue staining***					
1	P61983	14-3-3 protein gamma	↓3.8	Cytoplasm	Signal transducing adaptor protein
2	P62260	14-3-3 protein epsilon	↓2.6	Cytoplasm, nucleus	Signal transducing adaptor protein, brain
					development
3	P85969	Beta-soluble NSF attachment protein	↓1.6	Cell membrane	ER-Golgi transport, protein transport
4	P54311	Guanine nucleotide-binding protein	↓4.4	Cell membrane, cytoplasm	Signal transduction
		G(I)/G(S)/G(T) subunit beta-1			
5	P42123	L-lactate dehydrogenase B chain	↓1.9	Cytoplasm, cell membrane,	Pyruvate metabolic process
				mitochondrion	
6	P07335	Creatine kinase B-type	↓1.5	Cytoplasm	Brain development, creatine metabolism
7	Q6P9V9	Tubulin alpha-1B chain	↓2.1	Cytoplasm, cytoskeleton	Cell shape and movement
8	P63018	Heat shock cognate 71 kDa protein	↓2.1	Cell membrane, cytoplasm, nucleus	Protein folding, RNA processing, aging
9	P47942	Dihydropyrimidinase-related protein 2	↓1.8	Cytoplasm, cytoskeleton, cell	Brain development, neurogenesis, cell
				membrane	movement
10	P09117	Fructose-bisphosphate aldolase C	↓3.0	Cytoplasm	Energy metabolism (glycolysis), aging, apoptosis
11	P48500	Triosephosphate isomerase	↓2.2	Cytoplasm	Energy metabolism (glycolysis)

## Data Availability

Data are contained within the article.
